# mtPCDI: a machine learning-based prognostic model for prostate cancer recurrence

**DOI:** 10.3389/fgene.2024.1430565

**Published:** 2024-09-04

**Authors:** Guoliang Cheng, Junrong Xu, Honghua Wang, Jingzhao Chen, Liwei Huang, Zhi Rong Qian, Yong Fan

**Affiliations:** ^1^ Department of Urology Surgery, The Fourth People’s Hospital of Jinan, Jinan, Shandong, China; ^2^ Beidou Precision Medicine Institute, Guangzhou, China

**Keywords:** machine learning, targeted cancer therapy, prostate cancer, programmed cell death, mitochondrial activity, tumor immune microenvironment

## Abstract

**Background:**

This research seeks to formulate a prognostic model for forecasting prostate cancer recurrence by examining the interaction between mitochondrial function and programmed cell death (PCD).

**Methods:**

The research involved analyzing four gene expression datasets from The Cancer Genome Atlas (TCGA) and Gene Expression Omnibus (GEO) using univariate Cox regression. These analyses identified genes linked with mitochondrial function and PCD that correlate with recurrence prognosis. Various machine learning algorithms were then employed to construct an optimal predictive model.

**Results:**

A key outcome was the creation of a mitochondrial-related programmed cell death index (mtPCDI), which effectively predicts the prognosis of prostate cancer patients. It was observed that individuals with lower mtPCDI exhibited higher immune activity, correlating with better recurrence outcomes.

**Conclusion:**

The study demonstrates that mtPCDI can be used for personalized risk assessment and therapeutic decision-making, highlighting its clinical significance and providing insights into the biological processes affecting prostate cancer recurrence.

## 1 Introduction

Prostate cancer, a prevalent cancer affecting men, especially in older age categories, originates from the prostate gland and often progresses slowly, exhibiting few noticeable symptoms (Siegel et al., 2023). The development of this cancer is influenced by genetic, environmental, and lifestyle factors. Early detection is crucial for managing the disease and is primarily achieved by prostate-specific antigen (PSA) testing, which is also vital for tracking recurrence of the disease after treatment (Fenton et al., 2018). Treatment alternatives include active monitoring, surgery, radiotherapy, androgen deprivation therapy, chemotherapeutic treatments, targeted therapies, and immunotherapies. Recent advances in treatments and a deeper understanding of the disease’s molecular mechanisms have significantly improved patient outcomes (Nuhn et al., 2019; Subudhi et al., 2020; Rehman et al., 2023; Xie et al., 2023). However, outcomes can vary depending on the molecular properties of the tumors and individual patient differences.

PCD plays an important role in maintaining tissue stability by removing cells tare defective, excessive, or potentially cancerous through processes such as apoptosis, anoikis, and autophagy. This activity is crucial for preventing tumor formation by eliminating cells that could become cancerous. However, the interaction between PCD and tumors is complex, influencing tumor resistance, proliferation, and metastasis. In cancer therapies, harnessing PCD mechanisms to eradicate tumor cells is a common strategy. Nevertheless, a significant challenge arises as tumor cells may develop resistance to these treatments, for example, by enhancing autophagy, thereby complicating therapeutic outcomes (Galluzzi et al., 2018).

Additionally, mitochondria, essential organelles known as the “powerhouses of the cell,” are primarily responsible for ATP production and energy generation. They also play a crucial role in various biochemical processes including energy metabolism, cellular signaling, and the regulation of cell growth and death. Dysfunctions in mitochondrial functions, such as impaired respiratory capacity and morphological changes, are critical because the management of cell death is significantly impacted and cellular health and function are vitally maintained (Green et al., 2011; Bock and Tait, 2020; Wang et al., 2022; Vringer and Tait, 2023).

Mitochondria are critical in energy conversion and regulate a variety of cellular processes including PCD, which is essential for maintaining organismal stability. In prostate cancer, mitochondrial dysfunction significantly affects tumor cell survival by altering cell death pathways, thereby impacting disease progression and the effectiveness of treatments.

The normal growth and function of the prostate are heavily dependent on the androgen and androgen receptor (AR) pathway, which is also crucial in the development and progression of prostate cancer. Castration therapy, or Androgen Deprivation Therapy (ADT), halts the progression of prostate cancer by triggering programmed cell death and significantly shrinking prostate volume through the activation of intrinsic and extrinsic apoptotic pathways. Stress signals activate BH3-only proteins, initiating the intrinsic apoptotic pathway, which leads to the release and activation of pro-apoptotic proteins BAX and BAK. These proteins increase the permeability of the mitochondrial outer membrane (MOMP), resulting in the release of pro-apoptotic factors like cytochrome c. This process culminates in the formation of apoptotic bodies and the activation of a caspase cascade, ultimately causing cell apoptosis (Brenner and Mak, 2009; Choudhary et al., 2014; Tan et al., 2015; Ali and Kulik, 2021).

This study investigates into the effects of mitochondrial dysfunction and programmed cell death on recurrence-free survival (RFS) in prostate cancer patients. By leveraging multi-omics data and machine learning techniques, it aims to uncover the intricate relationships between mitochondrial dysfunction, programmed cell death, and the clinical features of prostate cancer. The objective is to identify precise molecular targets to develop personalized treatment strategies. Given the limited understanding of the interactions between mitochondrial dysfunction and PCD in prostate cancer, this research introduces the mtPCDI. This index is designed to predict treatment outcomes and prognosis, thereby aiding in the evaluation of clinical outcomes and the selection of optimal treatment approaches for prostate cancer patients.

## 2 Materials and methods

### 2.1 Data prepare

Clinical and transcriptomic datasets were collected from 895 patients using TCGA and GEO databases. The TCGAbiolinks R toolkit (Colaprico et al., 2016) was employed to retrieve 483 samples from the TCGA-PRAD cohort and the GEOquery R toolkit (Davis and Meltzer, 2007) to was utilized to procure 412 samples from four GEO cohorts: GSE116918 (248 samples), GSE54460 (100 samples), GSE70768 (19 samples), and GSE7079 (45 samples). All RNA-seq data were standardized to Transcripts Per Million (TPM), and potential batch effects in the GEO datasets were rectified utilizing using the “combat” method from the “sva” toolkit (Leek et al., 2012). The five cohorts were used to develop the recurrence prognosis model, with the TCGA cohort serving as the training set and the GEO cohort as the validation set. The GSE150368 cohort included pre- and post-treatment transcriptomic data from six individuals with locally advanced prostate cancer who received neoadjuvant androgen deprivation therapy (ADT). Additionally, data normalization was performed across all samples to ensure consistency in the analysis. Genomic analysis involved determining copy number variations using gistic2 applied to TCGA prostate cancer Masked Copy Number Segment files, and somatic mutations were sourced from TCGA prostate cancer mutect maf mutation files (Mermel et al., 2011). Mutation data were analysed using maftools (Mayakonda et al., 2018). Additionally, five types of genomic scores were integrated, based on the results of Thorsson V. (Thorsson et al., 2018). This holistic approach allowed for a thorough exploration of genomic alterations in prostate cancer.

### 2.2 Identification of prognostic mitochondrial-related and PCD-related genes

In a comprehensive study, 1,414 genes were identified as being associated with 18 types of programmed cell death (Zou et al., 2022). Additionally, 1,011 genes related to mitochondrial functions were extracted from the MitoCarta 3.0 database (Rath et al., 2021). Using the Wilcoxon test to analyze gene expression differences between prostate cancer tissues and normal tissues, significant differences were identified with a log2 fold change greater than one and an FDR below 0.001. The results were visualized using the “VennDiagram” software, showcasing differentially expressed genes involved in mitochondrial functions and PCD. Furthermore, co-expressed genes highly related to mitochondria and PCD were pinpointed in TCGA-PRAD samples through Spearman correlation analysis, with thresholds set at absolute correlation coefficients surpassing 0.6 and a *p*-value below 0.001.

### 2.3 Pathway activity analysis

Pathway activity scores (PAS) for TCGA prostate cancer samples were obtained from the GSCA database (Liu et al., 2023). Samples were stratified into high and low expression categories based on median gene expression values from the TCGA prostate cancer dataset. The Wilcoxon test compared PAS between these categories, considering a gene as potentially activating a pathway if its PAS was higher in the high-expression category. Statistical significance was determined with an FDR threshold of less than 0.05.

### 2.4 Tumor cell stemness

Data from the PCBC dataset were utilized, focusing on stem cells and their differentiated progenitors. Using the OCLR algorithm, a stemness index was derived and applied to transcriptomic expression data from the TCGA dataset, resulting in the development of TCGA Prostate Cancer mRNA Stemness Indices (mRNAsi) (Malta et al., 2018).

### 2.5 Prognostic model construction

30 models from seven distinct machine learning algorithms, including Lasso, GBM, Random Forest, Elastic Net, Stepwise Cox, Ridge, SuperPC were integrated. Their effectiveness was tested on four independent external datasets. Model accuracy was assessed using Harrell’s Consistency Index (C-index). The criteria for selecting the optimal model include two aspects. First, the C-index values of multiple cohorts have the highest consistency. Second, the average C-index value is the highest among these cohorts. And the best-performing model was identified (Liu et al., 2022). For larger prostate cancer (PRAD) cohorts exceeding 100 samples, individuals were stratified into high and low categories based on median scores of mtPCDI. In smaller cohorts of fewer than 50 samples, optimal cutoff points were determined using the survimer R package. Kaplan-Meier curves were utilized to explore the prognostic significance of recurrence-free survival, and the survivalROC R package was employed to generate calibration and receiver operating characteristic (ROC) curves, aiding in evaluating the prognostic performance of the models (Heagerty et al., 2000).

### 2.6 Biological function and pathway enrichment analysis

Various bioinformatics tools and databases were utilized to investigate the biological functions, pathways, and immune landscape associated with mtPCDI. Initially, hallmark cancer-associated signatures from the Molecular Signatures Database (MSigDB) were analyzed to discern their relevance to mtPCDI (Liberzon et al., 2015). Using the GSVA R package (Hanzelmann et al., 2013), scores were assigned to each tag, and differences in expression between varying levels of mtPCDI were identified via limma analysis, adhering to a significance threshold of a corrected *p*-value below 0.05 (Ritchie et al., 2015).

### 2.7 Immune microenvironment assessment

In the context of immune infiltration, several computational methods were employed to detail the immune landscape, supported by the use of single-sample gene set enrichment analysis (ssGSEA) targeting specific marker genes. Additionally, the expression of 123 immune-regulatory genes, which included factors of receptor, major histocompatibility complex, immunostimulatory factors, and chemokines was thoroughly examined (Charoentong et al., 2017). Further enrichment was achieved by incorporating signatures from the Tracking Tumor Immunophenotype (TIP) database (Xu et al., 2018), linked to the Cancer Immunity Cycle, and calculating the Tumor Inflammation Signature (TIS) based on 18 characteristic genes. Insights into immune functions were enriched by 13 signatures compiled from research by Bindea et al. (2013); Liu et al. (2018), while the immunedeconv R package integrated seven algorithms such as cibersort and mcp_counter to analyze immune cell infiltration using prostate cancer samples from TCGA (Sturm et al., 2019). Moreover, data concerning prostate cancer immune subtypes and multi-omics classifications were also derived from TCGA’s pan-cancer datasets, along with an Androgen Receptor (AR) score extracted from the Cancer Genome Atlas Research Network’s prostate cancer research (Cancer Genome Atlas Research, 2015). This multi-faceted approach not only enhanced the understanding of the molecular and immunological aspects of prostate cancer but also paved the way for potential targeted therapies.

### 2.8 Drug sensitivity analysis

In this research, the “oncoPredict” R package was employed to predict the IC50 of various drugs using TCGA prostate cancer expression data. Differences in IC50 between mtPCDI categories were analyzed using the Wilcoxon test (Maeser et al., 2021).

### 2.9 Construction of clinical nomograms

Multivariable Cox regression was utilized to pinpoint significant indicators of clinical recurrence at 1, 3, and 5 years, taking into account the mtPCDI score and other clinical factors. Significant predictors identified from the Cox regression analysis were used to develop prognostic nomograms, which were visualized by “regplot” package in R. The accuracy of these nomograms was evaluated by the generation of calibration curves with the “rms” package, and their clinical value was assessed by calculating their net benefits using the “ggDCA” package.

### 2.10 Statistical methods

Additionally, the Fisher test was applied to compare variable distributions across two categories, and Spearman correlation analysis was used to investigate relationships among variables. The influence of risk factors on survival outcomes was further examined through the Cox regression model, while Kaplan-Meier survival analysis was performed to evaluate these outcomes. All statistical analyses and scientific plotting were performed using R 4.2.1.

## 3 Results

**FIGURE 1:**
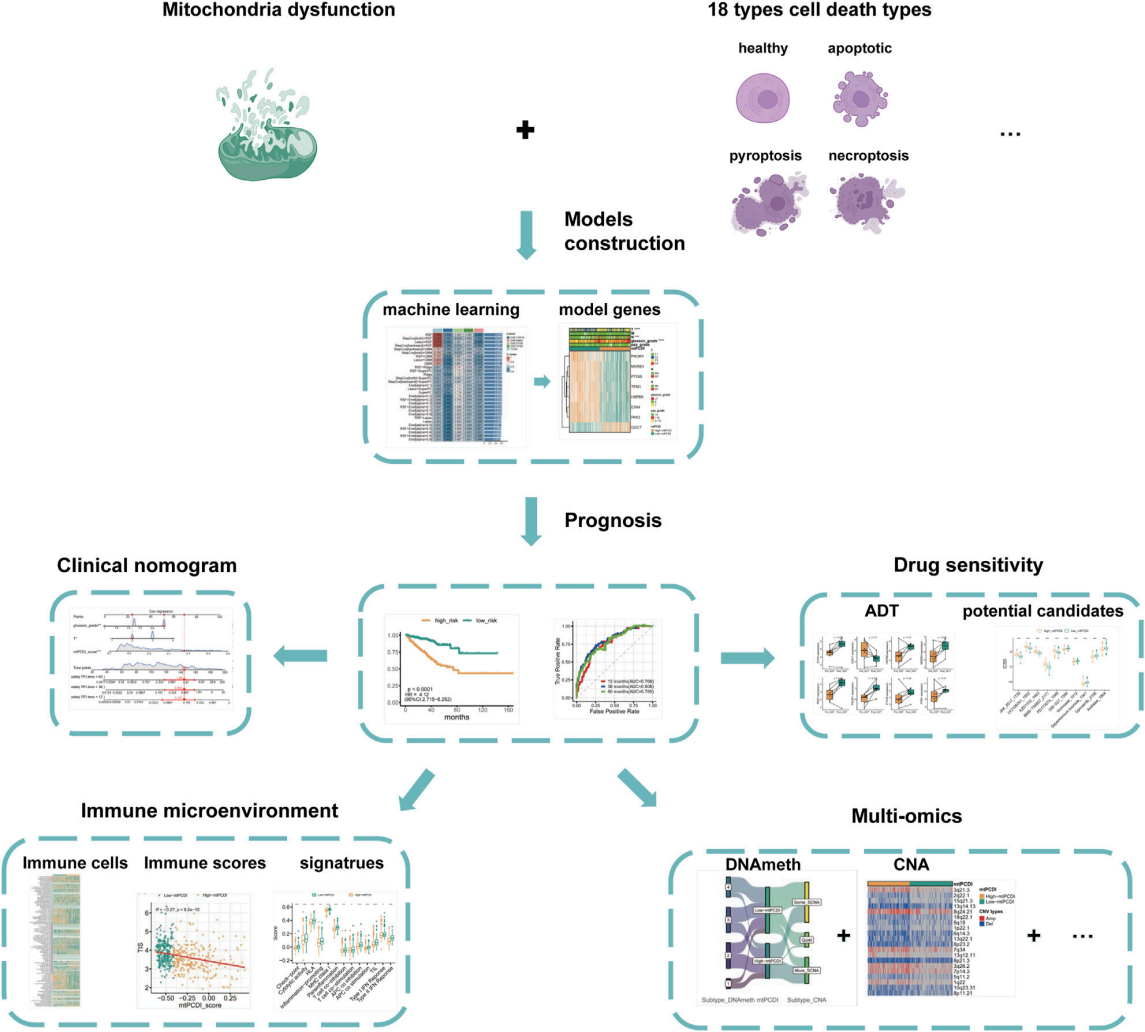
Analysis framework.

An overview of the research is presented in Figure 1. The basic information of the prostate cancer cohort is detailed in Supplementary Table S1, while the gene sets associated with programmed cell death and mitochondrial function used in the study are listed in Supplementary Table S2.

The study first identifies significantly differentially expressed genes by comparing normal and cancerous prostate tissues. Further, using Spearman correlation analysis, genes related to mitochondrial function and programmed cell death with high correlation coefficients and low *p*-values are screened. Univariate Cox regression analysis determines which of these co-expressed genes significantly impact the recurrence-free survival of prostate cancer patients. Utilizing machine learning algorithms, combined with TCGA-PRAD and external validation datasets, the mtPCDI model is developed, and key genes influencing RFS are identified. The predictive capability of the mtPCDI model is validated across multiple prostate cancer datasets by calculating AUC values to assess its accuracy. The gene expression patterns of patients with different mtPCDI scores are analyzed to explore their effects on the cell cycle, AR signaling pathway, mitochondrial dynamics, and other aspects. The genomic variations, including gene copy number, mutation frequency, and genetic instability, are compared among patients with different mtPCDI scores. The relationship between mtPCDI and tumor immune activity is investigated by analyzing immune cell infiltration and immune-related signaling pathways in patients with varying mtPCDI scores. The mtPCDI index has been attempted to be linked with multiple omics characteristics and the AR signaling pathway. By combining mtPCDI scores and clinicopathological features, a clinical nomogram is developed through multivariate Cox regression analysis to enhance the accuracy of prognosis prediction. Based on the mtPCDI model, the sensitivity of patients with different mtPCDI scores to specific drugs is assessed, providing a reference for personalized treatment.

### 3.1 Preliminary screening of mtPCDI regulators

**FIGURE 2:**
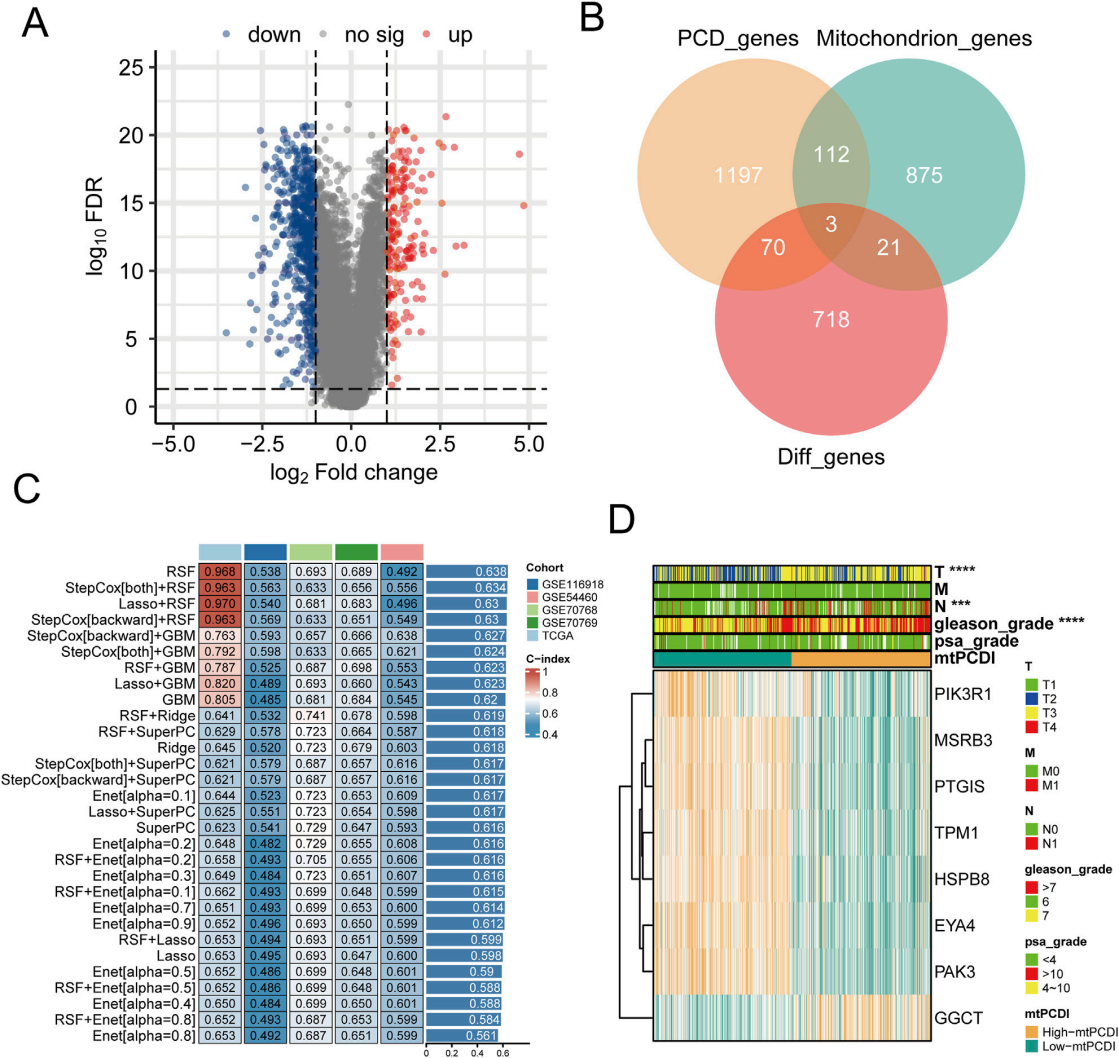
Prognostic model development and mtPCDI model **(A)** Volcano diagram depicting the gene expression differences between normal and cancerous prostate tissues. **(B)** Examination of differential gene expression associated with mitochondrial function and programmed cell death in prostate tumors versus normal tissue **(C)** Construction of prognostic models through the application of diverse machine learning algorithms. **(D)** Assessment of gene expression profiles within the GBM model stratified by mtPCDI categories.

Differential expression analysis highlighted 812 genes with significant expression differences between normal and cancerous prostate tissues (Figure 2A). This analysis also revealed distinct expression patterns for 24 mitochondrial function-related genes and 73 PCD-related genes (Figure 2B). Using Spearman co-expression analysis, the study identified 62 genes from these categories that had a correlation coefficient over 0.6 and a *p*-value below 0.001. Moreover, a univariate Cox regression analysis conducted on the TCGA-PRAD dataset indicated that 23 of these co-expressed genes significantly influenced RFS in PRAD patients. 30 different machine learning algorithms were employed to develop the mtPCDI, utilizing data from TCGA-PRAD and four external validation datasets. The most effective model combined the StepCox [backward] and GBM algorithms, pinpointing eight critical genes that affect RFS in PRAD patients (Figure 2C). Of these, seven genes were associated with longer RFS and one with shorter survival (Supplementary Table S3). Finally, the mtPCDI scores from the GBM model effectively distinguished patients into high and low risk categories, revealing substantial expression differences, mostly with genes expressed more in the low mtPCDI category (Figure 2D), suggesting a novel method for prognosis stratification in PRAD patients.

### 3.2 Prognostic analysis of the mtPCDI model

**FIGURE 3:**
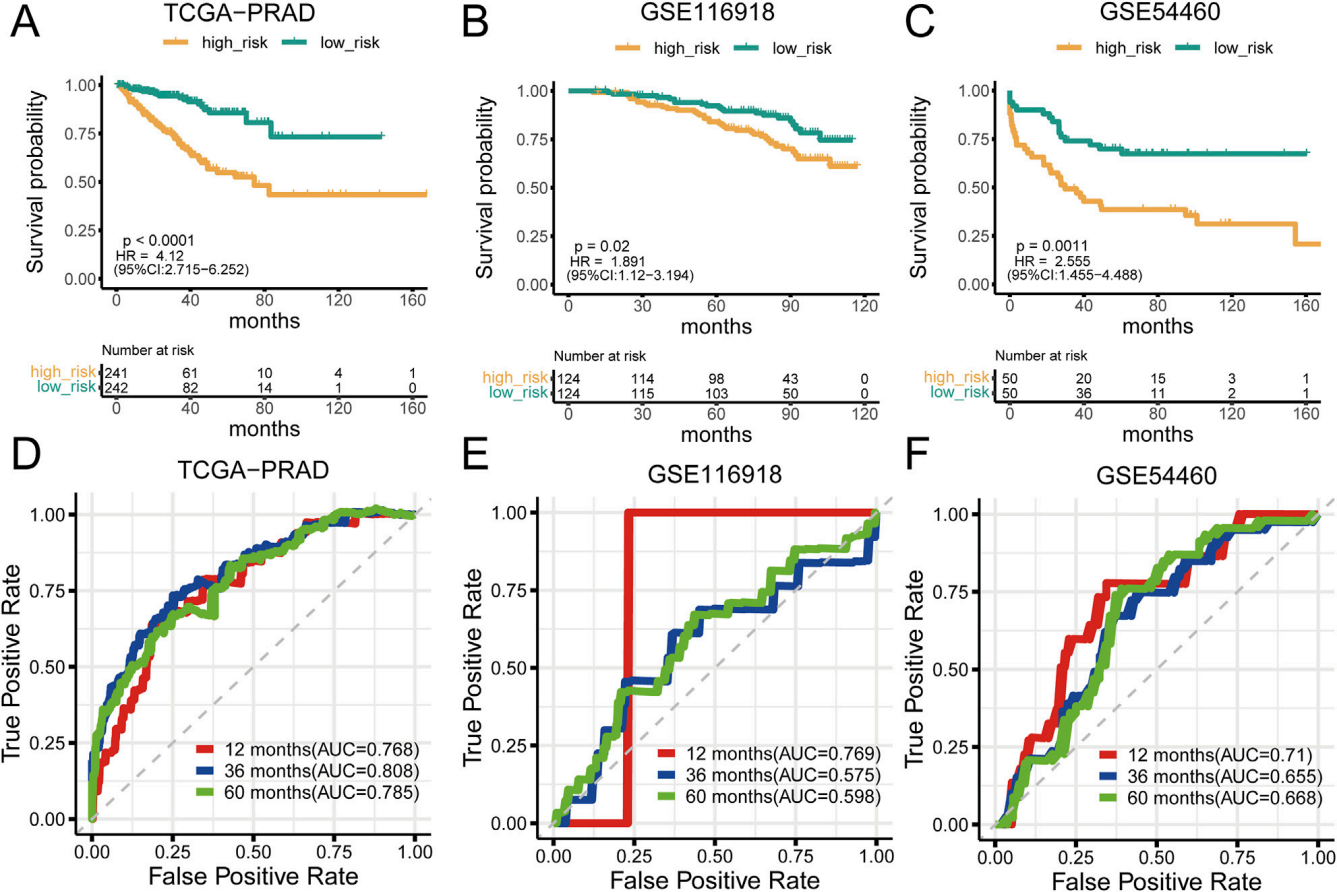
Prognostic significance of mtPCDI **(A–C)** RFS curves delineating different mtPCDI score categories within the PRAD TCGA, GSE116918, and GSE54460 cohorts. **(D–F)** ROC curves predicting RFS within 5 years using mtPCDI scores across the specified cohorts.

Analysis of prostate cancer datasets such as TCGA-PRAD, GSE116918, GSE54460, and GSE70768 has provided significant insights into mtPCDI scores. It has been observed that high mtPCDI scores are linked to shorter RFS times, as depicted in Figures 3A–C. Within these studies, the TCGA-PRAD cohort demonstrated robust Area Under the Curve (AUC) values of 0.768, 0.808, and 0.785 indicating strong predictive capabilities for 1-year, 3-year, and 5-year prostate cancer recurrence, respectively. For the GSE54460 cohort, AUC scores were 0.71, 0.655, 0.668, and for the GSE116918 cohort, AUC scores were 0.769,0.575 and 0.598 (Figures 3D–F). Furthermore, smaller datasets such as GSE70768 and GSE70769 reinforced the link between high mtPCDI and shorter RFS times, as referenced in Supplementary Figures S1A, 1B. And the AUC values were 0.83 and 0.625 respectively (Supplementary Figure S1). The GSE7079 dataset also showed consistent results. And the AUC values were 0.747, 0.719, and 0.72 (Supplementary Figure S1). These collective findings underscore the predictive significance of mtPCDI scores across various datasets, highlighting their potential utility in clinical assessments of prostate cancer prognosis.

### 3.3 Mechanism comparison between different mtPCDI categories

**FIGURE 4:**
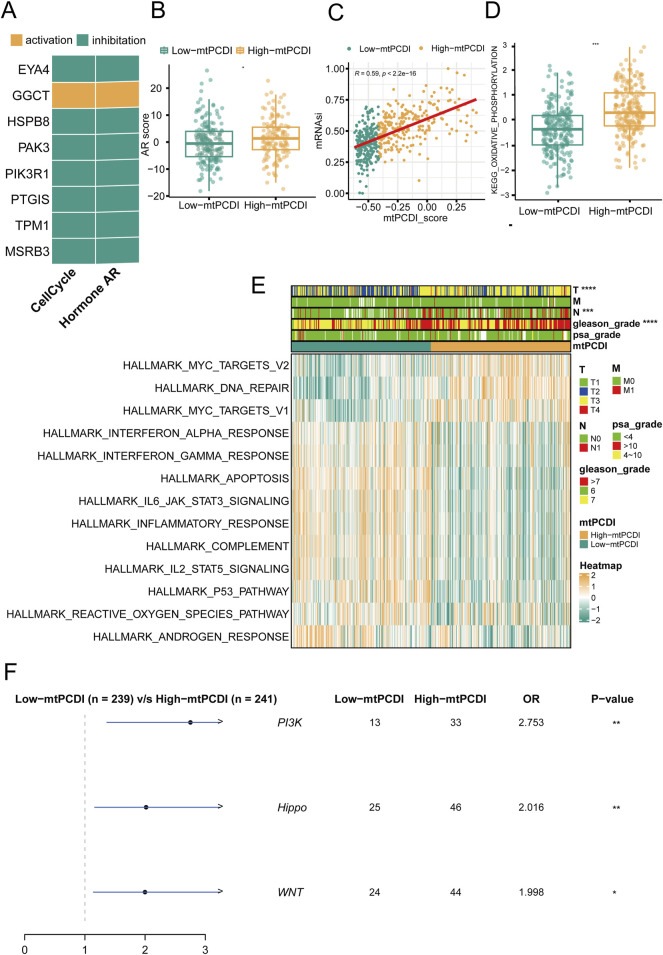
Pathway mechanisms across mtPCDI categories **(A)** Analysis of gene expression correlations with pathway activation levels. **(B)** Comparison of androgen receptor (AR) activity scores between high and low mtPCDI categories. Each box illustrates the quartile range of the data, with the median denoted by the horizontal line Statistical differences were assessed through the Wilcoxon test, as denoted by the significance levels. **(C)** Relationship between mtPCDI scores and tumor cell stemness metrics. **(D)** Differential analysis of mitochondrial metabolism-related KEGG enrichment pathway scores between high and low mtPCDI categories (***p < 0.001; **p < 0.01; *p < 0.05). **(E)** Heatmap representation of cancer-related pathway scores derived from gene expression data across mtPCDI categories. **(F)** Distinctions in mutation-driven pathway activities among mtPCDI categories.

In prostate cancer research, the expression patterns of model genes significantly influence cellular behaviors and disease outcomes. Seven of the eight model genes generally suppress cell cycle and AR (androgen receptor) signaling pathways when expressed at high levels, although GGCT stands out by potentially activating these pathways instead (Figure 4A). Regarding mitochondrial dynamics, patients with higher mtPCDI exhibit increased AR activity and higher tumor cell stemness scores, which are indicative of aggressive cancer traits (Figures 4B, C). In addition, the oxidative phosphorylation pathway score associated with mitochondrial function shows significant differences between the high and low mtPCDI categories. Patients in the low mtPCDI category have lower oxidative phosphorylation scores compared to those in the high mtPCDI category (Figure 4D). This high mtPCDI category also shows enhanced activity in the MYC target signaling pathway and DNA repair mechanisms, as determined by Gene Set Variation Analysis. Conversely, lower mtPCDI categories display more active immune-associated signaling pathways, including IL6 JAK STAT3 signaling and the Interferon Gamma response, Inflammatory response, and Apoptosis, suggesting different immune engagement (Figure 4E). Moreover, high mtPCDI categories have elevated somatic mutation frequencies in the PI3K, Hippo, and WNT signaling pathways, correlating with a more intricate genetic landscape and higher aggressiveness and progression of the disease (Figure 4F). This comprehensive analysis reveals a complex interplay between mitochondrial function, gene expression, and signaling pathways, underscoring their pivotal roles in the progression and characterization of prostate cancer.

### 3.4 Genomic comparison between different mtPCDI categories

**FIGURE 5:**
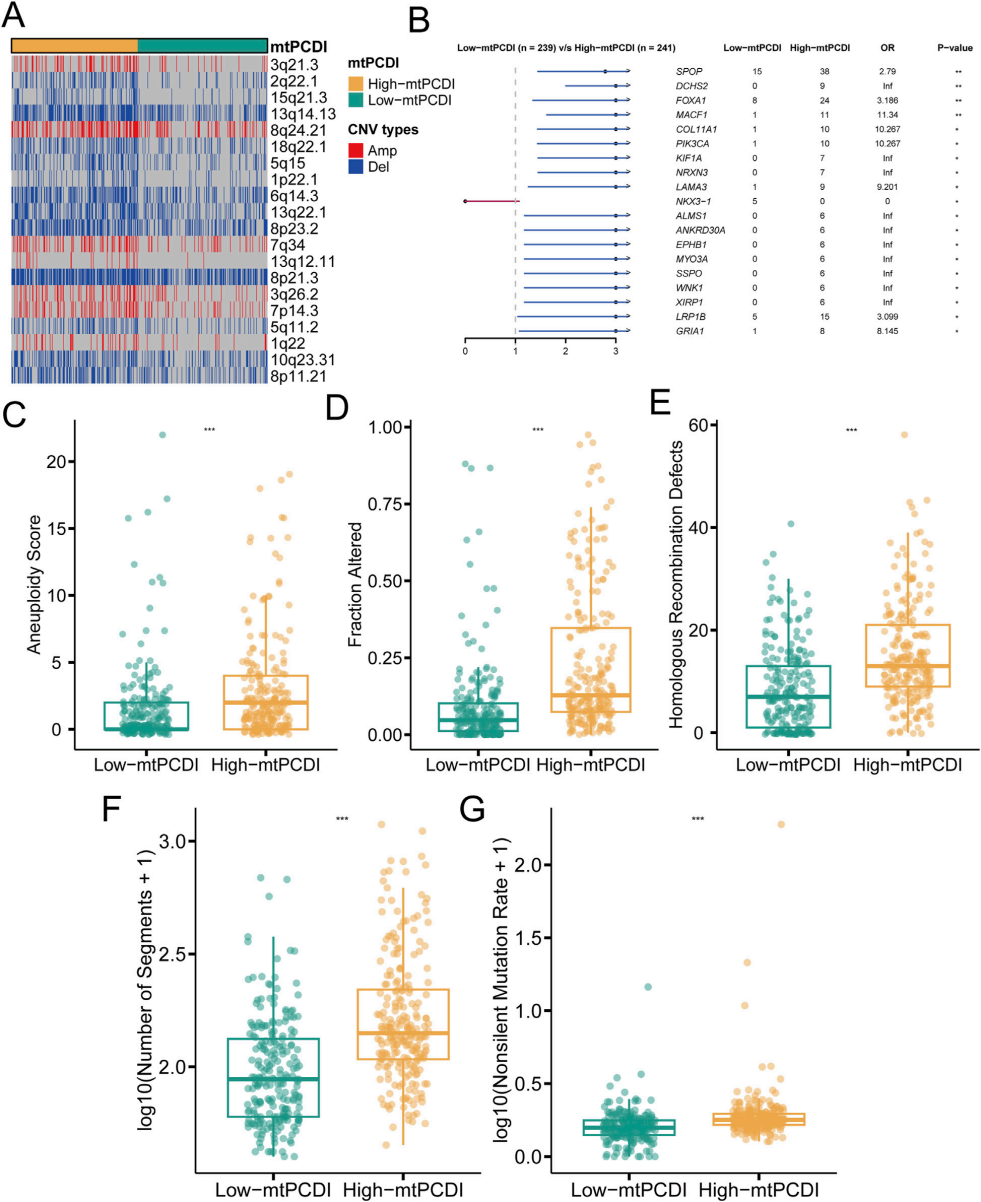
Genomic characteristics of mtPCDI categories **(A)** Heatmap illustrating segmental copy number variations within different mtPCDI categories. **(B)** Forest plot indicating differences in somatic mutation rates for specific genes across mtPCDI categories. **(C–G)** Comparative genomic analysis of various genomic scores between high and low mtPCDI score categories. Each box illustrates the quartile range of the data, with the median denoted by the horizontal line Statistical differences were assessed through the Wilcoxon test, as denoted by the significance levels (***p < 0.001; **p < 0.01; *p < 0.05).

In individuals with a high mtPCDI, distinct pattern in genetic alterations has been observed. Compared to those with a low mtPCDI, these individuals demonstrate an increased number of genetic copies at locus 8q24.21, while showing decreased copy numbers at loci 2q22.1, 5q11.2, and 5q15 (Figure 5A). Furthermore, the high mtPCDI category is associated with an higher occurrence of non-silent mutations and exhibits significantly elevated aneuploidy scores, indicating a greater degree of chromosomal instability. This category also displays increased values in homologous recombination defects and segments number, alongside indications of fraction altered. Statistical analysis confirms that these differences in aneuploidy scores, fraction altered, and homologous recombination defects are significant, each with a *p*-value of less than 0.001 (Figures 5C–G). Additionally, genes such as SPOP, DCHS2, FOXA1, MACF1, COL11A1, and PIK3CA show more frequent mutations in the high mtPCDI category (Figure 5B), suggesting different pathways of genetic instability between the two categories.

### 3.5 Immune microenvironment analysis of different mtPCDI categories

**FIGURE 6:**
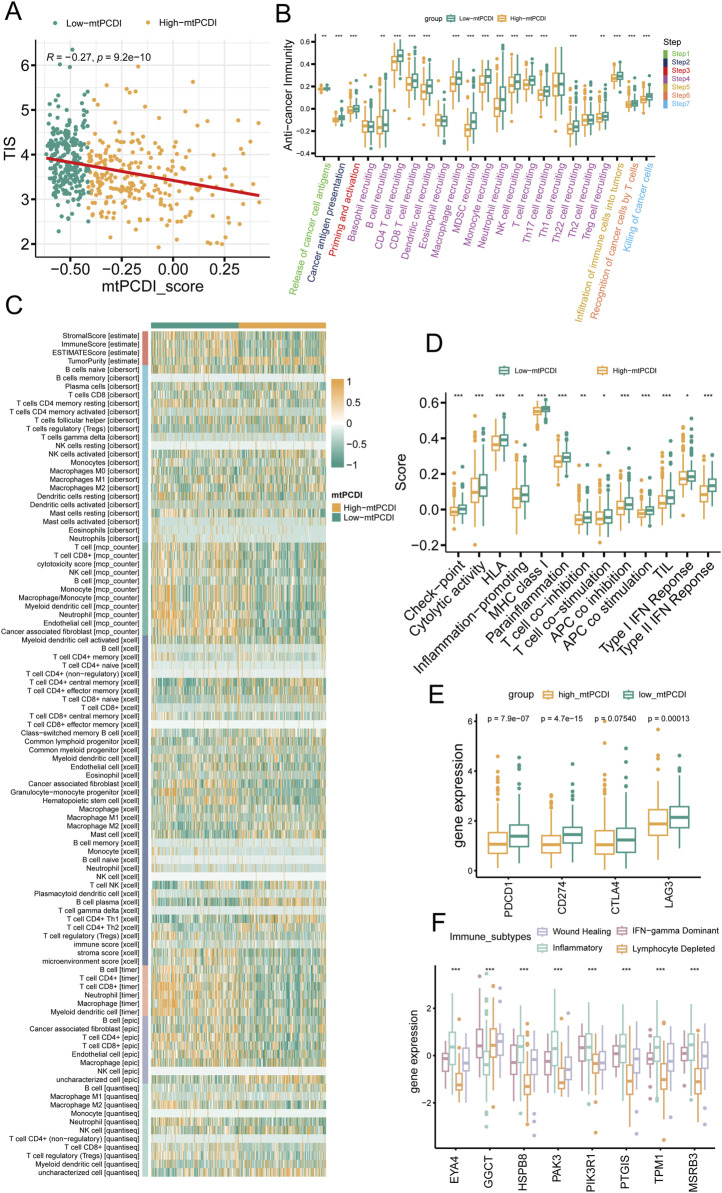
Immune microenvironment analysis by mtPCDI categories **(A)** Correlation of mtPCDI scores with the Tumor Inflammation Signature (TIS). **(B)** Evaluation of immune cycle scores across mtPCDI categories. **(C)** Heatmap integrating results from six immune cell analysis tools and ESTIMATE scores by mtPCDI category. **(D)** Variations in immune-related scores between high and low mtPCDI score categories. **(E)** Dissimilarities in prevalent immune checkpoint activities by mtPCDI category. **(F)** Expression variances of mtPCDI model genes within diverse immune subtypes. Each box illustrates the quartile range of the data, with the median denoted by the horizontal line Statistical differences were assessed through the Wilcoxon test, as denoted by the significance levels (***p < 0.001; **p < 0.01; *p < 0.05).

The research illustrates a negative relationship between the mtPCDI and the Tumor Inflammation Index (TIS), showing that lower mtPCDI scores are linked to enhanced tumor immune activity (Figure 6A). This relationship is highlighted by the progression of the tumor immune process through seven stages, where each stage showing higher activity scores in the category with lower mtPCDI category (Figure 6B). Among the six immune cell infiltration prediction software, the results of three are consistent: TIMER, EPIC, and MCP-counter. In these three software, B cells, CD4^+^ T cells, CD8^+^ T cells, NK cells, and macrophages all show consistent differences, which are important immune infiltrating cells. Other software also show a similar trend, with B cells, CD8^+^ T cells, and NK cells having higher infiltration abundance in the low mtPCDI category. Further validation is provided by the estimate algorithm, elevated immune and stromal scores, and reduced tumor purity in the low-mtPCDI category (Figure 6C). Additionally, there is a heightened expression of MHC class I, chemokine, receptors, cytokines, and immune checkpoint-related genes, alongside elevated responses in Type I and II interferon signaling (Figures 6D, E; Supplementary Figure S2). Notably, there are significant variations in the expression of mtPCDI model-related genes across different immune subtypes, with the inflammatory subtype showing the highest expression and the wound healing subtype the lowest (Figure 6F). These findings collectively suggest that the mtPCDI is a crucial marker for assessing the tumor immune microenvironment and could significantly influence personalized immune therapy strategies.

### 3.6 Construction of mtPCDI clinical nomogram

**FIGURE 7:**
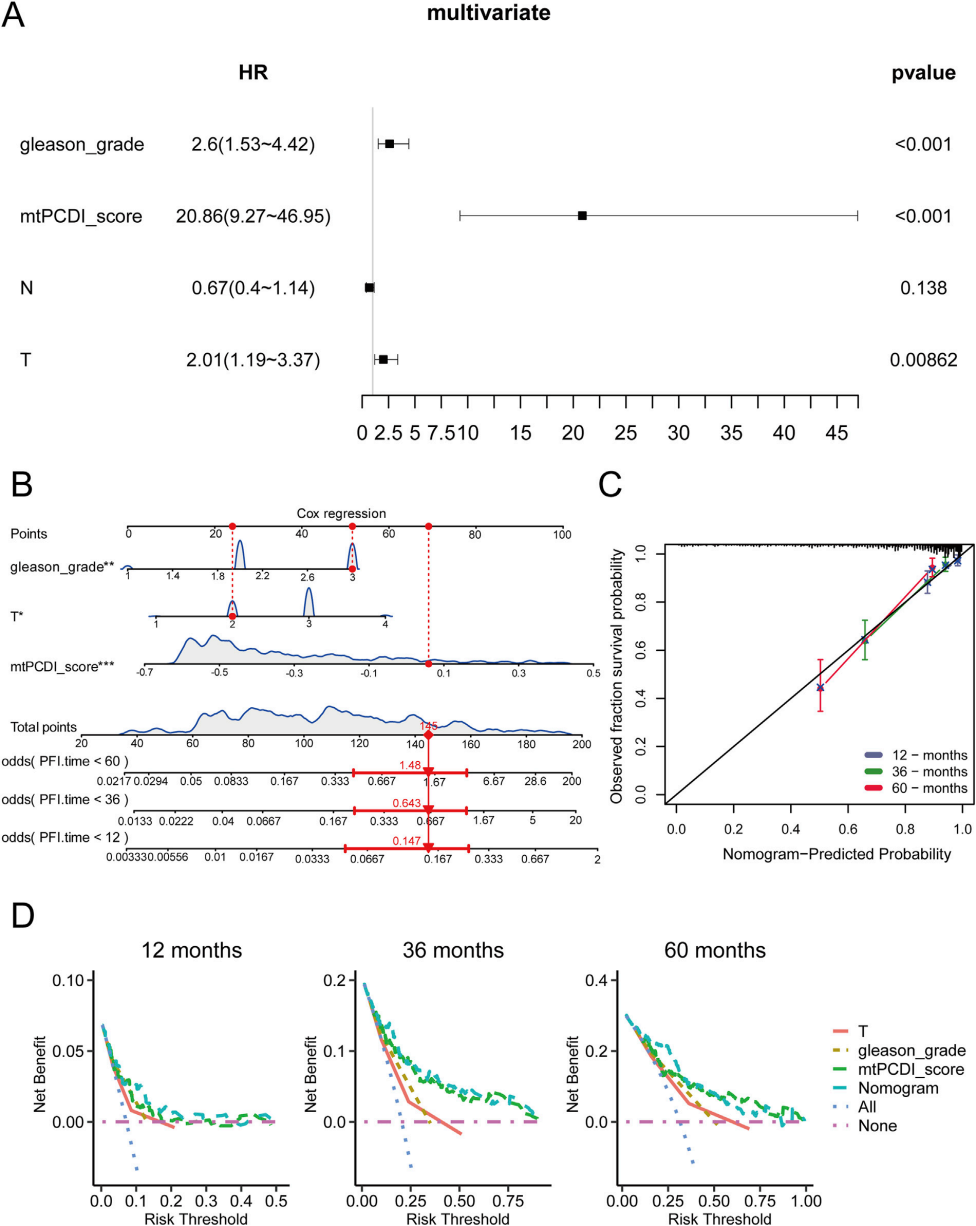
Independent prognostic value of mtPCDI in the immune context **(A)** Confirmation of mtPCDI score as an independent prognostic indicator. **(B)** Formulation of a multivariate clinical nomogram including the mtPCDI score. **(C)** Calibration plot for the clinical nomogram’s accuracy. **(D)** Decision curve analysis to compare the nomogram’s utility against other clinical predictors.

A multivariate Cox regression analysis was conducted to assess RFS in cancer patients, using the mtPCDI score along with clinical-pathological T and N staging, and Gleason grade. This analysis identified the Gleason grade, T stage, and particularly the mtPCDI score as significant prognostic factors, with the mtPCDI score emerging as an independent factor with a *p*-value below 0.001 (Figure 7A). To refine predictive capabilities further, a clinical nomogram incorporating these significant variables was developed, and its accuracy was confirmed by a calibration curve that closely matching the 45-degree diagonal, indicating precise predictions (Figures 7B, C). Over various time points, both the mtPCDI score and the nomogram exhibited consistent trends and demonstrated superior net benefits compared to using T stage and Gleason grade alone (Figure 7D). This enhancement in predictive accuracy highlights the utility of the mtPCDI score and the nomogram in improving clinical decision-making for predicting RFS.

### 3.7 Multivariate subtype correlation analysis in prostate cancer

**FIGURE 8:**
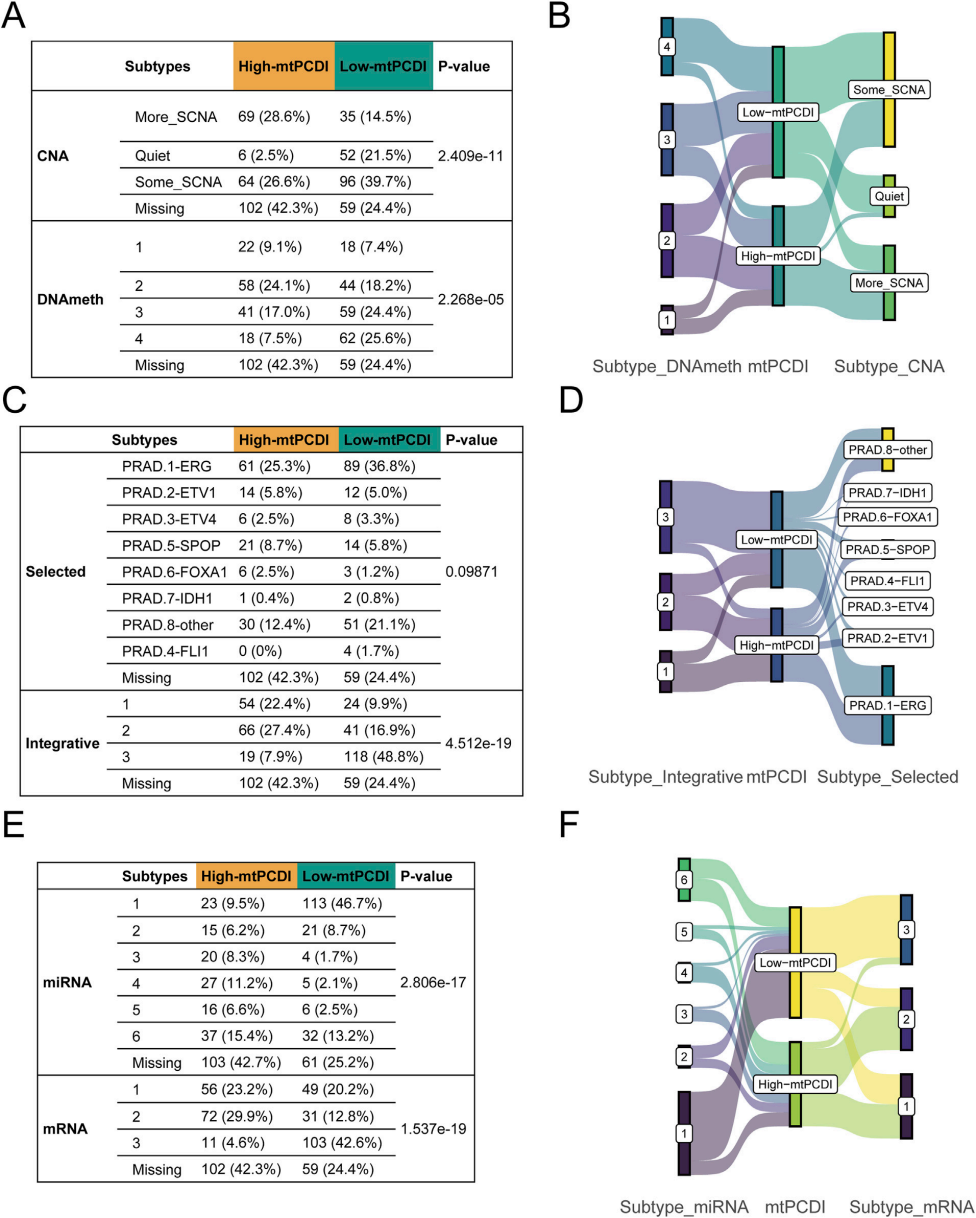
Multivariate subtype correlation with mtPCDI categories **(A–F)** Analysis encompassing Fisher’s tests and Sankey diagrams to explore the interrelations between mtPCDI categories and various genetic subtypes, including copy number variation, methylation, classical fusion mutations, integrative subtypes, and miRNA/mRNA subtypes.

Genetic and molecular characteristics showed significant variations between the high and low-mtPCDI categories. The high-mtPCDI category exhibited considerable genomic instability, characterized by increased copy number variations and methylation levels, with statistically significant *p*-values of 2.4096e-11 and 2.268e-5, respectively (Figures 8A, B).

Conversely, the low-mtPCDI category exhibited greater genomic stability, evidenced by lower methylation levels and a slightly higher incidence of specific fusion mutations such as ERG, EIV4, and FLI1, albeit with a less significant *p*-value of 0.09. Somatic mutations, including SPOP and FOXA1, were notably more prevalent in the high-mtPCDI category. In terms of intergrative subtypes, integrative subtypes 1 and 2, characterized by high tumor suppressor gene mutations, high methylation, low mRNA expression, and high copy number variations, were predominantly found in the high-mtPCDI category. In contrast, Subtype 3, which was more common in the low-mtPCDI category, showed lower methylation levels, the presence of some ERG fusions, higher mRNA expression, and fewer copy number variations, with a significant *p*-value of 4.512e-19 (Figures 8C, D).

Prominent differences in mRNA expression were also observed, with the High-mtPCDI category demonstrating overall lower expression levels overall, whereas the dominant subtype in the low-mtPCDI category showed higher expression levels, significantly marked by a *p*-value of 1.537e-19. Additionally, the low-mtPCDI category had a greater proportion of class 1 miRNA subtypes, which closely resemble the miRNA expression patterns of normal tissue, evidenced by a significant *p*-value of 2.806e-17. These findings underscore the distinct molecular and genetic landscapes of each mtPCDI category, suggesting potential specific targets for tailored therapeutic strategies (Figures 8E, F).

### 3.8 mtPCDI model drug sensitivity analysis in prostate cancer

**FIGURE 9:**
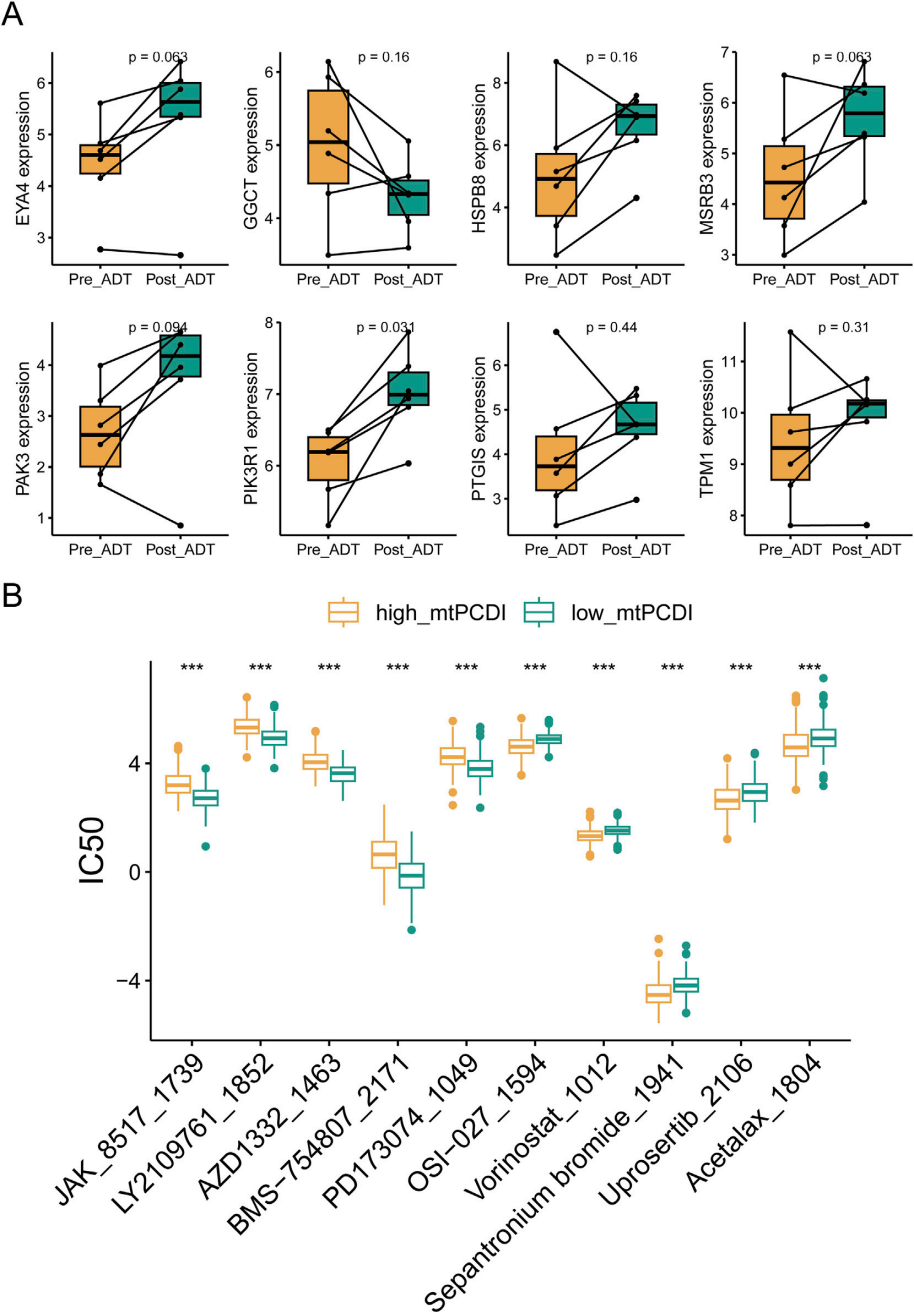
Drug sensitivity analysis by mtPCDI categories **(A)** Comparative gene expression analysis within the mtPCDI model pre- and post-androgen deprivation therapy (ADT). **(B)** The ten most significant differences in drug sensitivity between high and low mtPCDI score categories. Each box illustrates the quartile range of the data, with the median denoted by the horizontal line. Statistical differences were assessed through the Wilcoxon test, as denoted by the significance levels (***p < 0.001; **p < 0.01; *p < 0.05).

RNA sequencing revealed that ADT treatment led to an upregulation of cell death-related genes, except for GGCT, in lesion tissues. Post-treatment gene expression patterns resembled those of the low-risk category in the mtPCDI model (Figure 9A). The study identified differences in drug sensitivity between the high and low-mtPCDI categories, highlighting drugs like JAK_8517_1739, BMS−754,807_2171, and PD173074_1049 were more effective in patients with low mtPCDI scores. Conversely, drugs such as Vorinostat_1012, Sepantronium bromide_1941, and Uprosertib_2106 showed greater effectiveness in the high mtPCDI category (Figure 9B).

## 4 Discussion

The mtPCDI is an innovative prognostic model designed to predict recurrence risks in prostate cancer patients, serving as a pivotal tool in personalized medicine. This biomarker reflects the interactions between mitochondrial function and programmed cell death pathways, highlighting how mitochondrial dysfunction can lead to cancer recurrence by enabling cells to evade apoptosis. Higher mtPCDI values, indicating compromised mitochondrial function, are associated with poorer outcomes due to increased copy number variations, which potentially contribute to greater tumor heterogeneity and treatment resistance.

The mtPCDI index is being attempted to be linked with multiple omics characteristics. In prostate cancer research, several genetic and epigenetic factors significantly impact tumor behavior and patient prognosis. Chromosomal alterations, such as amplification at 8q24.21 and deletions at 5q11.2 and 5q15, crucially alter cellular functions (Liu et al., 2008; Chen et al., 2020). For example, mutations in the SPOP gene lead to increased stability of tumor-associated proteins like PTEN and enzymes involved in the PI3K/mTOR signaling pathway, enhancing cell proliferation. These mutations also prevent the breakdown of androgen receptor proteins, boosting AR signaling and thereby fostering tumor growth. Similarly, mutations in the FOXA1 gene activate AR signaling, promotes tumor growth, facilitating the epithelial-mesenchymal transition, and increasing tumor metastasis potential by inducing transcriptional changes that activate the WNT signaling pathway (Barbieri et al., 2012; Blattner et al., 2017).

Moreover, studying of methylation patterns reveals that higher methylation in certain prostate cancer categories can lead to gene silencing, affecting tumor progression and patient outcomes. Variations in miRNA expression further delineate distinct regulatory mechanisms influencing tumor development and cell death pathways. Understanding these complex interactions and integrating multi-omic data, including methylation patterns and miRNA profiles, can significantly refine personalized treatment strategies. For instance, patients with higher methylation levels might respond better to epigenetic therapies with demethylating agents, suggesting that a tailored approach to treatment could benefit for those with significant genetic alterations, potentially requiring more aggressive management (Okano et al., 1999; Lyko and Brown, 2005; Jones and Baylin, 2007).

Mitochondria are crucial to the development of prostate cancer, influencing energy bioenergetics, programmed cell death, and immune cell function regulation. Dysfunction in these organelles can alter the tumor microenvironment by modifying inflammatory signals and cytokine release, thus affecting immune cell behavior. This is quantifiable through the Mitochondrial Programmed Cell Death Index (mtPCDI), which assesses mitochondrial functionality and cell death status. A lower mtPCDI, signifying healthier mitochondria and normal cell death processes, is associated with enhanced immune activity and improved responses to immune therapies, such as checkpoint inhibitors (Xie et al., 2023).

In addition, the connection between the androgen receptor (AR) pathway in prostate cancer, mitochondrial dysfunction, and programmed cell death requires further investigation. Furthermore, AR is integral to the development and progression of prostate cancer, with many cancer cells relying on AR signaling for survival and growth. ADT, which targets AR activity, is a common treatment approach; however not all patients respond well, and some eventually develop resistance, leading to treatment failure and recurrence. AR not only impacts cellular processes like energy metabolism and apoptosis but also mitochondrial function, playing a crucial role in the cancer’s behavior and treatment outcomes (Marquez-Jurado et al., 2018; Bajpai et al., 2019; Ahmad and Newell-Fugate, 2022).

By integrating assessments of mitochondrial function, programmed cell death (via mtPCDI), and AR activity, clinicians might gain comprehensive biomarker insights. This integrated approach could enhance predictions of patient responses to ADT and their recurrence risks. Typically, high AR activity correlates with more aggressive cancer types and poorer outcomes. Tumors maintaining high AR activity post-ADT often resist treatment and continue progressing. Some cancers adapt to low-androgen environments by altering AR signaling, which supports their survival and proliferation. Evaluating both mtPCDI and AR activity allow for more refined patient classification and assist in selecting aggressive treatments, such as novel AR inhibitors or combination therapies, particularly for patients exhibiting high levels of both indicators (Parker et al., 2020; Labriola et al., 2021).

Mitochondrial function plays a pivotal role in cellular bioenergetics and the regulation of cell death, potentially influencing redox states and energy balance, and leading to apoptosis. This is particularly relevant in prostate cancer treatments, where dysfunctional mitochondria can affect the stability and activity of the AR. Enhancing mitochondrial function could potentially increase the effectiveness of ADT in treating prostate cancer. Therefore, future research should focus on the interactions between mitochondrial PC-induced death (mtPCDI) and AR activity, especially in relation to treatment resistance and disease recurrence. It is also crucial to validate mitochondrial and AR-related biomarkers to predict treatment outcomes and guide therapeutic decisions (Baumgartner et al., 2024).

This study has certain limitations. In terms of sample size, the number of samples in this study was constrained by the clinical information available in public databases, preventing a larger-scale sample study. Due to the heterogeneity of prostate cancer, whether this model is universally applicable for predicting prostate cancer recurrence still requires further construction of clinical follow-up data and additional research. Furthermore, commonly used PSA levels and Gleason scores in prostate cancer could be considered in combination with the mtPCDI index as a predictive model for patient recurrence in the future.

Furthermore, utilizing multi-omics approaches could greatly enhance treatment strategies, particularly in immunotherapy for prostate cancer. Resistance issues in treatments such as Enzalutamide, used for castration-resistant prostate cancer (CRPC), might be addressed by investigating JAK2 inhibitors like JAK_8517_1739, which target the growth and resistance pathways activated by Enzalutamide (Udhane et al., 2020). Additionally, Vorinostat, a histone deacetylase inhibitor, is currently under clinical trials to assess its effectiveness against metastatic CRPC, especially for its synergistic effects when combined with the mTOR inhibitor Temsirolimus. Vorinostat is also being evaluated in clinical trials (e.g., NCT00589472, NCT01174199, NCT00330161) for potential benefits in conjunction with radical prostatectomy and androgen deprivation therapy.

## 5 Conclusion

The mtPCDI signature, initially developed using the TCGA-PRAD cohort, was subsequently validated across four external cohorts, demonstrating its superiority over existing clinical models as a prognostic marker. Its predictive accuracy remained robust even after adjusting for potential confounding factors. Further research explored the relationship between mtPCDI, immunomodulators, and the tumor microenvironment, providing valuable insights and laying the groundwork for more detailed future studies in these areas.

## Data Availability

Publicly available datasets were analyzed in this study. The TCGA data can be found here: https://portal.gdc.cancer.gov/projects/TCGA-PRAD, and the GEO data can be found here: https://www.ncbi.nlm.nih.gov/geo/, with accession numbers GSE116918, GSE54460, GSE70768, GSE7079, and GSE150368.
