# Supplementary material for: mtPCDI: a machine learning-based prognostic model for prostate cancer recurrence
Source: Front Genet. 2024 Sep 4;15:1430565. doi: 10.3389/fgene.2024.1430565 (PMC11408181; doi:10.3389/fgene.2024.1430565)
Supplement: Supplementary file 2 [file Image1.pdf]

Supplementary Material

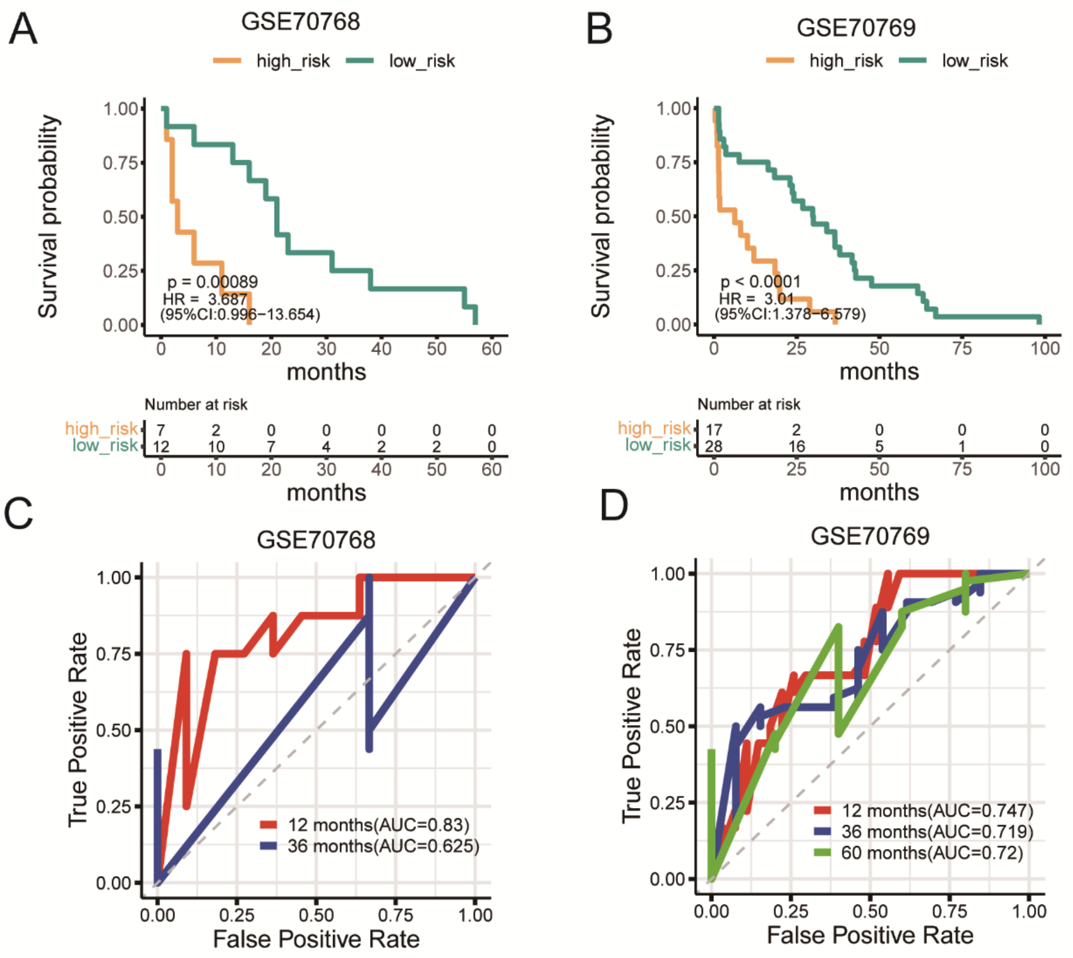

Figure S1 Prognostic Significance of mtPCDI in GSE70768 and GSE70769 cohort. (A-B) Kaplan-Meier survival curves comparing high vs. low mtPCDI score groups within GSE70768 and GSE70769 cohorts. (C-D) ROC curves for predicting 1-year, 3-year, and 5-year RFS using mtPCDI scores across GSE70768 and GSE70769 cohorts.

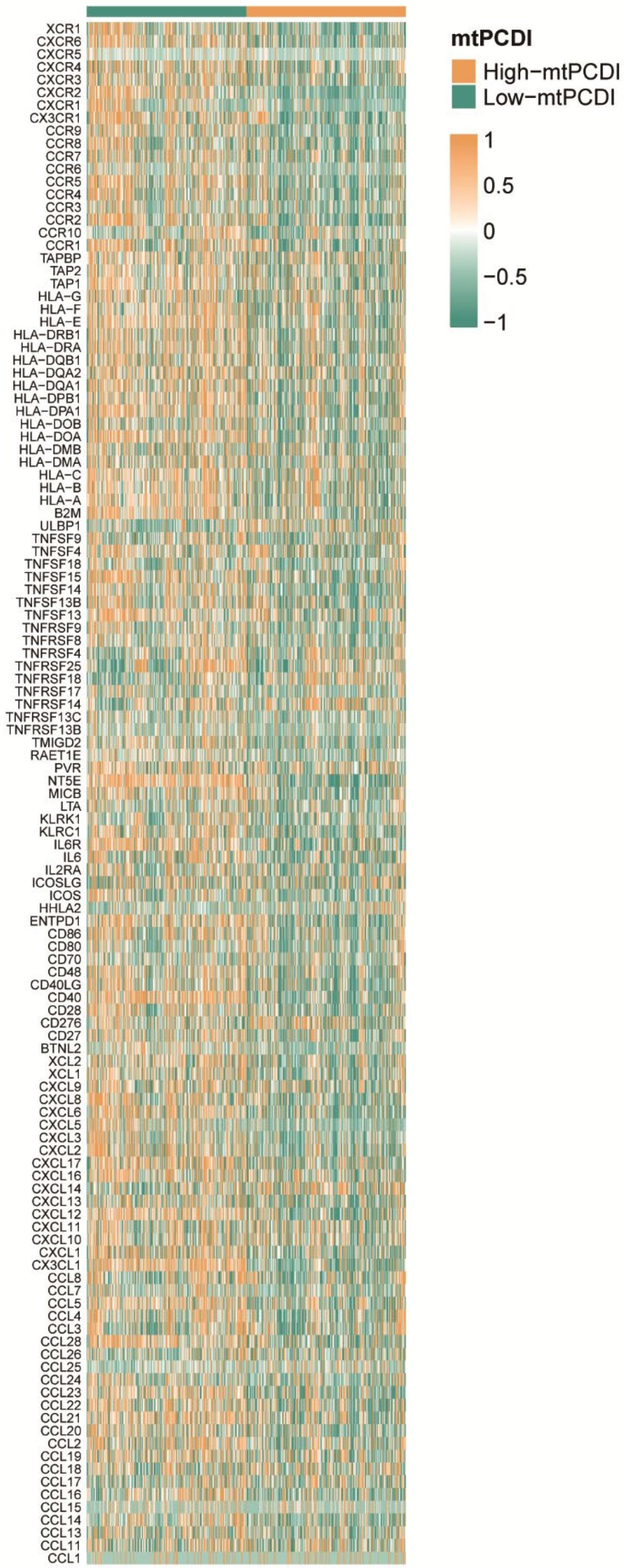

Figure S2 Expression heatmap of immune modulator-related genes
